# Hypertension and type 2 diabetes: What family physicians can do to improve control of blood pressure - an observational study

**DOI:** 10.1186/1471-2296-12-86

**Published:** 2011-08-11

**Authors:** Wayne Putnam, Beverley Lawson, Farokh Buhariwalla, Mary Goodfellow, Rose Anne Goodine, Jennifer Hall, Kendrick Lacey, Ian MacDonald, Frederick I Burge, Nandini Natarajan, Ingrid Sketris, Beth Mann, Peggy Dunbar, Kristine Van Aarsen, Marshall S Godwin

**Affiliations:** 1 Department of Family Medicine, Dalhousie University, Oxford St., Halifax, NS, B3H 4R2, Canada; 2 Community-based physician; 3 College of Pharmacy, Dalhousie University, Oxford St., Halifax, NS, B3H 4R2, Canada; 4 Department of Medicine, Dalhousie University, Oxford St., Halifax, NS, B3H 4R2, Canada; 5 Diabetes Care Program of Nova Scotia, South Park St., Halifax, NS, B3H 2Y9, Canada; 6 Discipline of Family Medicine, Memorial University of Newfoundland, PO Box 4200, St. John's, NL, A1C 5S7, Canada

## Abstract

**Background:**

The prevalence of type 2 diabetes is rising, and most of these patients also have hypertension, substantially increasing the risk of cardiovascular morbidity and mortality. The majority of these patients do not reach target blood pressure levels for a wide variety of reasons. When a literature review provided no clear focus for action when patients are not at target, we initiated a study to identify characteristics of patients and providers associated with achieving target BP levels in community-based practice.

**Methods:**

We conducted a practice- based, cross-sectional observational and mailed survey study. The setting was the practices of 27 family physicians and nurse practitioners in 3 eastern provinces in Canada. The participants were all patients with type 2 diabetes who could understand English, were able to give consent, and would be available for follow-up for more than one year. Data were collected from each patient's medical record and from each patient and physician/nurse practitioner by mailed survey. Our main outcome measures were overall blood pressure at target (< 130/80), systolic blood pressure at target, and diastolic blood pressure at target. Analysis included initial descriptive statistics, logistic regression models, and multivariate regression using hierarchical nonlinear modeling (HNLM).

**Results:**

Fifty-four percent were at target for both systolic and diastolic pressures. Sixty-two percent were at systolic target, and 79% were at diastolic target. Patients who reported eating food low in salt had higher odds of reaching target blood pressure. Similarly, patients reporting low adherence to their medication regimen had lower odds of reaching target blood pressure.

**Conclusions:**

When primary care health professionals are dealing with blood pressures above target in a patient with type 2 diabetes, they should pay particular attention to two factors. They should inquire about dietary salt intake, strongly emphasize the importance of reduction, and refer for detailed counseling if necessary. Similarly, they should inquire about adherence to the medication regimen, and employ a variety of patient-oriented strategies to improve adherence.

## Background

The age-standardized prevalence of type 2 diabetes in Canada rose by a relative 21% from 2003 to 2007 to 6.2% overall [1]. Most of these patients with diabetes also have hypertension, a modifiable risk factor for heart disease. In a baseline phase preceding this study, 78.7% of type 2 diabetes patients in family practices in the Maritime Provinces had been diagnosed with hypertension [2], using routine office sphygmomanometers. This is higher than one other comparable Canadian study (63%) [3], but similar to a second (75.8%) [4]. As comorbidities, hypertension and diabetes substantially increase the risk of cardiovascular morbidity and mortality [5].

Although effective treatments exist for hypertension, most patients with type 2 diabetes do not reach target blood pressures. In the earlier baseline phase, only 27.1% of patients were at target (< 130/80) [2], within the range of 13 - 36% reported at target in other studies in community settings [6-10].

A review of the literature revealed a wide array of patient, physician, and health system factors contributing to the success or failure in reaching target BP goals. Many, such as patient age [4,11-17] and sex [4,11,13,16,18-20] and physician age [12] and sex [12,14] are not modifiable. Health system factors such as access to drug insurance plans have been cited by physicians as a major facilitator of care of patients with diabetes [21], but that too is not amenable to action on the part of the individual physician. Among the most frequently cited reasons for poor control of blood pressure in hypertensive patients is a lack of adequate drug treatment, with calls for a more aggressive approach [22,23]. Our baseline data had shown that patients were already taking an average of 2.5 antihypertensive medications [2], close to the 2.9 found necessary to reach a target of less than 130/80 in an American study [18].

The literature review provided no clear guidance to our team of eight community family physicians that had collected the baseline phase data regarding where to focus their attention when patients are not at target blood pressures. With the support of university colleagues, they initiated a study to identify characteristics of patients and providers associated with achieving target BP levels in patients with type 2 diabetes in typical community-based practice. This paper will outline which characteristics were found to be significant and provide suggestions for increased attention in office practice.

## Methods

This practice-based, cross-sectional study which included both observational and mailed survey components was conducted in family physician and nurse practitioner practices in the Maritime Family Practice Research Network in 3 eastern provinces in Canada. The study was first approved by our institutional Research Ethics Board (CD-2004-221); it was subsequently approved by six more Research Ethics Boards with jurisdiction over participating practices. Data for this phase of our study was collected between January 2008 and June 2010. All participants signed informed consent forms.

### Subjects

Potential participants included all patients of each practice with diagnoses of type 2 diabetes and hypertension who could understand English, were able to give consent, and were expected to be available for follow-up for more than one year. Patients were included if they had their most recent BP measurement taken with a BpTRU™ within the past six months in order to relate that pressure with the results of the patient survey. Because antihypertensive drug prescribing was one of our study variables, only patients taking those medications were included in the analysis.

### Data sources and measures

Data was obtained from three sources: the patient's medical record, a self-administered survey mailed to each patient and a survey completed by each participating provider. Measures (potential predictor and outcome variables) from all sources were guided by a modification of the Andersen conceptual model of health service utilization [24] and included a variety of individual patient (population) characteristics and contextual system and provider factors (Table 1).

**Table 1:** Andersen's Conceptual Model of Health Service Utilization adapted to investigate BP outcome

ENVIRONMENT →		POPULATION CHARACTERISTICS →			HEALTH BEHAVIOUR →	OUTCOMES
	**Health care** **system**	**Predisposing characteristics**	**Enabling Resources**	**Need**	**Use of Health Services**	**Evaluated health status**

**Level 1** **Individual **Patient factors		**Demographics** -Age -Gender -Ethnicity **Social Structure** -Education -Marital status	**Personal/family** -Private drug insurance	**Evaluated need** -Duration of hypertension -Duration of diabetes -Body mass index -HbA1c -HDL -LDL -Renal Status -Co-morbidity **Perceived need** -Bayliss' Comorbidity Index	**Antihypertensive drug intensity** **Health care system** -Diabetes Education Attendance **Personal health practices** -Medication Adherence (Morisky score) -Blood Pressure Self-Monitoring -Smoking -Physical activity -Diet -Alcohol Consumption	*Measured blood pressure (BP)* -Overall BP at target -Systolic BP at target -Diastolic BP at target
	
**Level 2** **Contextual** Physician/ Practice/ factors	**Province**		**Provider Characteristics** -Gender -Years in Family Medicine -Payment type -Practice type -Practice nurse (Y/N) -Urban/Rural			

Clinical data relevant to the diagnosis and management of type 2 diabetes and hypertension were extracted from each patient's medical record. Individual level patient characteristics included predisposing characteristics (age, gender) and factors associated with the identification of evaluated need such as the durations of diabetes and hypertension, targeted clinical factors (HbA1C equal to or less than 7.0%, LDL-CHOL < 2.5 mmol/L, and Total CHOL:HDL-CHOL ratio < 4.0) [25], renal status, and a co-morbidity index. Health service use from the medical record was represented by the intensity of antihypertensive drug prescribing. For each patient, details pertaining to all prescribed antihypertensive medications currently being taken at the time of BP recording were extracted. Antihypertensive drug classes included in the CHEP recommendations [26,27] were then used in the calculation of prescribing drug "intensity" through the adaptation of an approach first described by Menard et al. [28]. Briefly, for each patient, the total daily dose prescribed for each antihypertensive medication was divided by the upper limit of the range of doses usually effective for hypertension as listed in the 2006 *Compendium of Pharmaceuticals and Specialties *[29]. The sum of "intensity" scores for each drug was then calculated.

The main outcomes of interest, or evaluated health status, were overall BP at target, systolic blood pressure (SBP) at target and diastolic blood pressure (DBP) at target. BP readings extracted from the patient medical record were the most recent measures taken with an automated oscillometric machine (BpTRU™). The BpTRU™ readings were done according to a protocol provided with the machine, using the average of the second through fifth readings. Blood pressure at target was defined as less than 130/80 mmHg, consistent with the CHEP recommendations [26,27]. Details of clinical data extracted may be found in an earlier paper reporting the first phase of the study [2].

Information pertaining to patients not normally found in the medical record was obtained directly from them using a survey questionnaire developed for this study. Survey administration was by mail and followed a modified Dillman's method [30]. Each eligible patient was mailed an initial package containing a personalized letter of invitation, information about the study, the questionnaire and a post-paid return envelope. A follow-up reminder was sent to all who had not responded within five weeks after the initial invitation. Survey materials sent to patients were mailed from their provider's office. The patient survey solicited information regarding additional patient characteristics (predisposing characteristics, enabling resources, perceived need) and health behaviours (use of health services, personal health practices) (Table 1). To aid in the capture of some of this information, two validated, reliable scales were included: the Morisky Medication Adherence Scale, a four-item self-report adherence measure to evaluate medication adherence in hypertensive patients [31], and a comorbidity assessment instrument developed by E. A. Bayliss to estimate the number of comorbid conditions and patient's perceived disease burden from these conditions (a "comorbidity index") [32].

To account for clustering of patients within provider practices across the three provinces, contextual information pertaining to the practice and the system they operate within were collected through a survey mailed to each participating provider using the same method as that for the patient survey. Items in this provider survey solicited responses regarding provider demographics and practice information.

### Analysis

Following initial descriptive statistics, bivariate regression models were used to evaluate the crude association between each potential predictor variable and all outcomes of interest. This was followed by multivariate analysis where hierarchical nonlinear modeling (HNLM) was utilized in order to account for the clustering of patients associated with a provider. Predictors found to be significant at p ≤ 0.2 in the bivariate analyses were considered for inclusion in the hierarchical multivariate modeling. For the hierarchical analyses, all predictors were tested for random or fixed slopes and accounted for in the regression equations as required.

A manual stepwise procedure was used for the addition of individual predictors to build the model. Individual level patient predictors (Level 1) were tested first, followed by contextual system and provider predictors (Level 2). Predictors were considered statistically significant in the final model with a p-value ≤ 0.05.

HLM version 6.03 [33] was used for HNLM analyses. SAS version 9.1 [34] was used for all other analyses.

## Results

In total, 656 patients met the inclusion criterion of this study and were asked to complete the self-administered survey. Of these eligible patients, 588 completed the patient survey for a response rate of 89.6%. Eighteen respondents were removed from analyses due to missing critical information, such as incomplete survey information from their provider or antihypertensive medications not being prescribed. This resulted in a final cohort of 570 for analysis. These 570 patients had been recruited by 27 providers who represented a diverse group of family physicians (n = 25) and nurse practitioners (n = 2) from urban and rural settings, and various practice types and payment methods (Table 2). Only 11 or 1.9% of patients were associated with a nurse practitioner practice. Most patients (67.2%) came from practices in which virtually all eligible patients had been approached for participation. There were no statistically significant differences between patients in all practices for age, gender, blood pressure at target or antihypertensive drug intensity score.

**Table 2:** Contextual System and Provider Characteristics (n = 27)

CATEGORICAL VARIABLES	Physician Frequency (%)	Overall Number of Patients (%)
***System Characteristics***		

**PROVINCE**		
New Brunswick	10 (37.04)	191 (33.51)
Nova Scotia	16 (59.26)	348 (61.05)
Prince Edward Island	1 (3.70)	31 (5.44)

***Provider Characteristics***		

**GENDER**		
Male	13 (48.15)	415 (72.81)
Female	14 (51.85)	155 (27.19)

**MEAN YEARS IN PRACTICE (SD)**	20.37 (10.52)	---------------

**PROVIDER TYPE**		
Family Physician	25 (92.59)	559 (98.07)
Nurse Practitioner	2 (7.41)	11 (1.93)

**PRACTICE SETTING**		
Urban	16 (59.26)	303 (53.16)
Rural	11 (40.74)	267 (46.84)

**PAYMENT TYPE**		
Fee for Service	12 (44.44)	415 (72.81)
Other	15 (55.56)	155 (27.19)

**PRACTICE TYPE**		
Private office/clinic (excluding free standing walk-in clinic)	12 (44.44)	464 (81.40)
Community Clinic, Health Centre or Hospital	8 (29.63)	89 (15.61)
Academic Health Sciences Centre	7 (25.93)	17 (2.98)

**PRACTICE NURSE**		
No	8 (29.63)	280 (49.12)
Yes	19 (70.37)	290 (50.88)

Individual patient characteristics are summarized in Table 3. Overall 54.4% were at target for overall BP (both SBP and DBP), 62.1% were at SBP target, and 79.3% were at DBP target.

**Table 3:** Patient Individual Level Characteristics (n = 570)

*PREDISPOSING CHARACTERISTICS*		
***Demographics***	**Frequency (%)**	**Number at BP Target (%)**

AGE		
< 55 years	82 (14.39)	38 (46.34)
55-64 years	178 (31.23)	107 (60.11)
65-74 years	187 (32.81)	102 (54.55)
75 + years	123 (21.58)	63 (51.22)

GENDER		
Male	295 (51.75)	163 (55.25)
Female	275 (48.25)	147 (53.45

ETHNICITY		
White	488 (85.61)	265 (54.30)
Other	14 (2.46)	9 (64.29)
Unknown	68 (11.93)	36 (52.94)

***Social Structure***		

EDUCATION		
Grade 8-11	261 (45.79)	145 (55.56)
Completed High School	100 (17.54)	49 (49.00)
More Education than High School	187 (32.81)	101 (54.01)
Unknown Education	22 (3.86)	15 (68.18)

MARITAL STATUS		
Married/Common Law	385 (67.54)	211 (54.81)
Single/Separated/Widowed/Divorced	185 (32.46)	99 (53.51)

***ENABLING RESOURCES***		

***Personal/Family***		

PATIENT INSURANCE		
No	63 (11.21)	36 (57.14)
Yes	499 (88.79)	268 (53.71)

***NEED***		

***Evaluated Need (Clinical)***	**Frequency (%)**	

YEARS SINCE HYPERTENSION DIAGNOSIS		
In the past 5 years	126 (22.11)	64 (50.79)
5 to 10 years	163 (28.60)	102 (62.58)
Over 10 years	277 (48.60)	142 (51.26)
I do not remember	4 (0.70)	2 (50.00)

YEARS SINCE DIABETES DIAGNOSIS		
In the past 5 years	164 (28.77)	86 (52.44)
5 to 10 years	161 (28.25)	98 (60.87)
Over 10 years	237 (41.58)	122 (51.48)
I do not remember	8 (1.40)	4 (50.00)

GLYCEMIC CONTROL		
Controlled (HbA1c ≤ 7)	316 (56.13)	177 (56.01)
Uncontrolled (HbA1c > 7)	247 (43.87)	127 (51.42)

LIPIDS: LDL-CHOL		
Controlled (LDLc < 2.5)	408 (72.47)	229 (56.13)
Uncontrolled (LDLc ≥ 2.5)	155 (27.53)	78 (50.32)

LIPIDS: HDL-CHOL		
Controlled (Ratio TG:HDL < 4)	373 (65.67)	208 (55.76)
Uncontrolled (Ratio TG:HDL ≥ 4)	195 (34.33)	101 (51.79)

RENAL STATUS		
Missing	64 (11.23)	24 (37.50)
Normal	335 (58.77)	198 (59.10)
Microalbuminuria	83 (14.56)	51 (61.45)
Overt Nephropathy	88 (15.44)	37 (42.05)

***Perceived Need (Self-Report)***	**Frequency (%)**	

BAYLISS MEAN DISEASE COUNT (SD)	7.88 (4.56)	

***USE OF HEALTH SERVICES***		

MEAN INTENSITY OF ANTIHYPERTENSIVE MEDICTIONS (SD)	1.84 (1.01)	

***Health Care System***		

DIABETES EDUCATION CLINIC ATTENDANCE (PAST YEAR)		
Zero Visits	386 (67.72)	205 (53.11)
One visit	95 (16.67)	55 (57.89)
Two or More Visits	89 (15.61)	50 (56.18)

***Personal Health Practices***		

**MEDICATION ADHERENCE**		
High Adherence	404 (70.88)	221 (54.70)
Medium Adherence	110 (19.30)	55 (50.00)
Low Adherence	9 (1.58)	3 (33.33)
Missing	47 (8.25)	31 (65.96)

**BLOOD PRESSURE SELF-MONITORING**		
No	224 (39.30)	131 (58.48)
Sometimes	241 (42.28)	134 (55.60)
Often	105 (18.42)	45 (42.86)

**SMOKING**		
Current	62 (10.88)	35 (56.45)
Past	253 (44.39)	144 (56.92)
Never	225 (39.47)	119 (52.89)
Unknown	30 (5.26)	12 (40.00)

**PHYSICAL ACTIVITY**		
Not able to do physical activity	106 (18.86)	61 (57.55)
Once a week or less	143 (25.44)	74 (51.75)
2-3 times per week	157 (27.94)	89 (56.69)
4 or more times per week	156 (27.76)	82 (52.56)

**EATING FOODS LOW IN SALT**		
No	138 (24.34)	63 (45.65)
Yes	429 (75.66)	244 (56.88)

**ALCOHOL**		
Non or Occasional Drinker	392 (68.77)	210 (53.57)
Mild Drinker	164 (28.77)	92 (56.10)
Heavy Drinker	14 (2.46)	8 (57.14)

***EVALUATED HEALTH STATUS***	**Frequency (%)**	

**OVERALL BLOOD PRESSURE AT TARGET**		
No	260 (45.61)	
Yes	310 (54.39)	

**SYSTOLIC BLOOD PRESSURE AT TARGET**		
No	216 (37.89)	
Yes	354 (62.11)	

**MEAN SYSTOLIC BLOOD PRESSURE (SD)**	126.06 (15.51)	

**DIASTOLIC BLOOD PRESSURE AT TARGET**		
No	118 (20.70)	
Yes	452 (79.30)	

**MEAN DIASTOLIC BLOOD PRESSURE (SD)**	70.96 (9.94)	

NOTE: Sum of participants for each variable may not add to n = 570 due to missing responses.

Table 4 summarizes the results of the final multivariate analysis and shows a variety of statistically significant predictors of reaching target pressures. No predisposing or personal/family patient characteristics were significantly associated with attaining overall target BP. Two evaluated need characteristics (duration of diagnosis of hypertension, renal status) and three personal health practices (salt intake, medication adherence, BP self-monitoring) were significantly associated. Compared to patients diagnosed with hypertension over the past five years, those with a five to ten year duration of diagnosis of hypertension had greater chance of reaching overall targeted BP (adjusted odd ratio [AOR] = 1.62; 95% confidence interval [CI] = 1.07-2.44). Lower odds of reaching target BP was associated with patients diagnosed with overt nephropathy compared to those with a normal renal status (AOR = 0.48; 95%CI = 0.33-0.69). Eating food low in salt was associated with greater odds of reaching target BP (AOR = 1.74; 95% CI = 1.25-2.41). Low adherence to medication regimen (versus high) as reported on the Morisky Medication Adherence Scale was associated with lower odds of reaching target BP (AOR = 0.29; 95% CI = 0.09-0.86) as was the patient self-monitoring their BP (often versus no, AOR = 0.51; 95% 0.33-0.79).

**Table 4:** Statistically Significant Predictors for Reaching Target Blood Pressure

	OR (95% CL)		
**VARIABLE**	**BP at Target** **(< 130/80 mmHg)**	**Systolic BP at Target** **(< 130 mmHg)**	**Diastolic BP at Target** **(< 80 mmHg)**

**INDIVIDUAL LEVEL-1 PATIENT FACTORS**			

***Predisposing characteristics ***			

**AGE**			
< 55 years		---------------	---------------
55-64 years		0.93 (0.54-1.60)	1.87 (0.88-3.89)
65-74 years		0.62 (0.32-1.18)	3.56 (1.67-7.56)*
75 + years		0.48 (0.25-0.96)*	6.12 (2.66-14.12)*

**GENDER**			
Male			---------------
Female			2.09 (1.03-4.25)*

***Evaluated Need***			

**YEARS SINCE HYPERTENSION DIAGNOSIS**			
In the past 5 years	---------------		
5 to 10 years ago	1.62 (1.07-2.44)*		
Over 10 years ago	1.04 (0.67-1.60)		
I do not remember	1.12 (0.25-4.96)		

**LIPIDS: LDL-CHOL**			
Uncontrolled			---------------
Controlled			1.68 (1.14-2.49)*

**RENAL STATUS**			
Missing	0.52 (0.28-1.01)	0.45 (0.23-0.87)*	
Normal	---------------	---------------	
Microalbuminuria	1.10 (0.70-1.72)	1.28 (0.79-2.07)	
Overt Nephropathy	0.48 (0.33-0.69)*	0.48 (0.31-0.76)*	

***Personal Health Practices***			

**MEDICATION ADHERENCE**			
High Adherence	---------------	---------------	
Medium Adherence	0.72 (0.50-1.04)	0.80 (0.54-1.19)	
Low Adherence	0.29 (0.09-0.86)*	0.34 (0.12-0.96)*	
Missing	1.65 (0.79-3.43)	1.87 (0.92-3.82)	

**BLOOD PRESSURE SELF-MONITORING**			
No	---------------	---------------	
Sometimes	0.87 (0.58-1.30)	0.79 (0.53-1.18)	
Often	0.51 (0.33-0.79)*	0.47 (0.28-0.80)*	

**EAT FOODS LOW IN SALT**			
No	---------------		---------------
Yes	1.74 (1.25-2.41)*		2.35 (1.49-3.70)*

**NO CONTEXTUAL PHYSICIAN OR SYSTEM LEVEL-2 FACTORS WERE SIGNIFICANT IN THE HLM MODEL**			

	**OR (95% CL)**		

**VARIABLE**	**BP at Target** **(< 130/80 mmHg)**	**Systolic BP at Target** **(< 130 mmHg)**	**Diastolic BP at Target** **(< 80 mmHg)**

**INDIVIDUAL LEVEL-1 PATIENT FACTORS**			

***Predisposing characteristics ***			

**AGE**			
< 55 years		---------------	---------------
55-64 years		0.93 (0.54-1.60)	1.87 (0.88-3.89)
65-74 years		0.62 (0.32-1.18)	3.56 (1.67-7.56)*
75 + years		0.48 (0.25-0.96)*	6.12 (2.66-14.12)*

**GENDER**			
Male			---------------
Female			2.09 (1.03-4.25)*

***Evaluated Need***			

**YEARS SINCE HYPERTENSION DIAGNOSIS**			
In the past 5 years	---------------		
5 to 10 years ago	1.62 (1.07-2.44)*		
Over 10 years ago	1.04 (0.67-1.60)		
I do not remember	1.12 (0.25-4.96)		

**LIPIDS: LDL-CHOL**			
Uncontrolled			---------------
Controlled			1.68 (1.14-2.49)*

**RENAL STATUS**			
Missing	0.52 (0.28-1.01)	0.45 (0.23-0.87)*	
Normal	---------------	---------------	
Microalbuminuria	1.10 (0.70-1.72)	1.28 (0.79-2.07)	
Overt Nephropathy	0.48 (0.33-0.69)*	0.48 (0.31-0.76)*	

***Personal Health Practices***			

**MEDICATION ADHERENCE**			
High Adherence	---------------	---------------	
Medium Adherence	0.72 (0.50-1.04)	0.80 (0.54-1.19)	
Low Adherence	0.29 (0.09-0.86)*	0.34 (0.12-0.96)*	
Missing	1.65 (0.79-3.43)	1.87 (0.92-3.82)	

**BLOOD PRESSURE SELF-MONITORING**			
No	---------------	---------------	
Sometimes	0.87 (0.58-1.30)	0.79 (0.53-1.18)	
Often	0.51 (0.33-0.79)*	0.47 (0.28-0.80)*	

**EAT FOODS LOW IN SALT**			
No	---------------		---------------
Yes	1.74 (1.25-2.41)*		2.35 (1.49-3.70)*

**NO CONTEXTUAL PHYSICIAN OR SYSTEM LEVEL-2 FACTORS WERE SIGNIFICANT IN THE HLM MODEL**			

NOTE: Only variables found to be statistically significant in the final hierarchical models are included.

* Statistically significant

Factors significantly associated with reaching SBP target included a predisposing characteristic (age), an evaluated need factor (renal status) and personal health practices (medication adherence and BP self-monitoring). All were associated with lower odds of attaining SBP target. Age greater than 75 years was associated with lower odds for reaching target as were patients diagnosed with overt nephropathy, with low medication adherence and often self-monitoring their BP.

The attainment of DBP target was significantly associated with age and gender (predisposing characteristics), controlled low-density lipoprotein cholesterol (LDL-CHOL) (an evaluated need) and salt intake (a personal health practice). Female patients had twice the odds of being at DBP target as males (AOR = 2.09; 95% CI = 1.03-4.25) while patients 65 years of age and older were more than three times as likely as patients < 55 years. Also associated with greater odds of attaining DBP target were patients with controlled LDL-CHOL (AOR = 1.68; 95% CI = 1.14-2.49) and those reporting eating foods low in salt (AOR 2.35; 95% CI 1.49-3.70).

Contextual factors pertaining to the system or providers (level 2) did not add significantly to the final multivariate models. However there was significant variability in BP at target across providers, which justified the use of the multi-level method.

## Discussion

Of the 570 patients included in this analysis, 54.4% had achieved both SBP and DBP targets (< 130/80), much higher than the 27.1% reported in the baseline phase and comparable to a large study in Ontario, Canada, which also used BpTRU™ measurements (50.3%) [7]. Two potential explanations for this might be 1) more appropriate treatment consistent with CHEP recommendations [35,36], and 2) the use of automated BP machine which reduces white coat effect [37,38].

The remainder of the discussion will focus on three significant factors found by our study that potentially can be modified by interventions of health practitioners caring for hypertension. Patients who reported low medication adherence and self-monitoring of blood pressure were less likely to have achieved overall BP target levels while patients eating foods low in salt were more likely. These factors can be influenced by a wide array of health care providers.

Our results are in keeping with other studies that report an association between medication adherence and blood pressure control. In a study by Casson et al., poor adherence was associated with increases in systolic blood pressure when patients were monitored over an 18 month period [39]. Morris et al. reported that non adherent patients had higher systolic and diastolic blood pressures compared to adherent patients [40], and a meta-analysis of six studies by DiMatteo et al. showed that patients adherent to hypertensive medication had three times the odds of having better overall blood pressure control than patients who were non-adherent [41]. In the original study validating the Morisky Medication Adherence Scale, 75% of patients who reported high adherence had their BP under adequate control compared to 47% of patients who reported low adherence [31].

A review of strategies to enhance hypertensive medication adherence found that no single intervention has emerged as superior to others. It is recommended that a patient-centered approach that is tailored to overcome specific patient barriers may be the best strategy to improve adherence to hypertensive medication [42]. A variety of steps can be taken in primary care that have been shown to be effective in improving adherence: decreasing the number of daily doses [43], giving written directions and ensuring that patients understand the treatment regimen [44], home [45], or self [46] monitoring, and using medications with fewer side effects [47]. It has also been shown that pharmacist intervention can reduce SBP [48], and a recent study has reported that family physicians believe that more communication with pharmacists on the adherence issue could improve it [49]. A helpful table of strategies has been posted on the web by CHEP http://hypertension.ca/chep/therapy-tables/#table 5. Further research could clarify which of these strategies is most effective in improving adherence and lowering blood pressure.

The association of self-monitoring of blood pressure with a lower likelihood of achieving BP targets contrasts with much of the literature on the topic. Bray et al. reported on a review of 25 randomized controlled trials that had been published by 2009 that included self-monitoring and self-management of blood pressure as an intervention [50]. Twelve of those studies had a co-intervention with the self-monitoring; examples include patient education, contact with a health professional such as a nurse or pharmacist, phone contact, home visit, or telemetry. They concluded that self-monitoring "reduces blood pressure by a small but significant amount" [50]. Subsequently, McManus et al. demonstrated that telemonitoring and self-management (self-monitoring plus self-titration of antihypertensive medications) were effective in controlling systolic BP [51]. Bosworth et al. conducted a randomized trial that included self-monitoring as one arm of the study. They found that self-monitoring combined with a tailored behavioral telephone intervention "improved BP control, systolic BP, and diastolic BP at 24 months relative to usual care" but the differences with self-monitoring alone were not significant [52].

Our study was an observational study, with no explicit intervention for self-monitoring. The data presented came from the patient survey response to the question "Do you check your blood pressure outside of your doctor's office?" Response options were "No," "Sometimes," or "Often." Those who responded "often" were less likely to be at target (overall and SBP). We have no evidence to determine how many patients were advised to self-monitor by their doctor or nurse practitioner as a component of "usual care," or how many chose to do it themselves at home or in a setting such as a pharmacy. There was no structured co-intervention. Our study design did not provide us with evidence to explain the apparent difference with previous literature. We have speculated that individuals who were not at target might be more likely to be concerned about their BP and therefore check it between office visits. It may also be that, without a co-intervention, self-monitoring alone offers a small [50] to no [52] advantage.

Our finding that patients who report a low salt diet are more likely to be at overall target and diastolic BPs comes as no surprise and gives support to current guidelines [27]. It strongly suggests where health care providers could focus their attention when dealing with patients whose pressures are still higher than desired. Discussion of salt restriction, on an equal footing with the medications they must take, will emphasize to the patient the importance of this restriction. A verbal "prescription" to specifically decrease salt can accompany written prescriptions for medications. A referral to, and conversation with, a nutritionist or diabetes educator regarding counseling on diet and sodium reduction, could ensure that this is treated as a high priority by the patient.

Our "real world" community practice setting, in rural and urban locations with a variety of available resources and supports for patients, is a strength of this study. A small number of academic practices (10) contributed only 8% of the patient cohort. None of the practices had a special focus on diabetes; all were broad primary care practices of physicians and nurse practitioners interested in contributing to research. As well, our high patient survey response rate (89.6%) reinforces the value of our data and enhances the generalizability of our findings. An additional strength is the use of hierarchical nonlinear modeling which allowed us to control for the clustering of patients within practices and the variance between providers.

One of the limitations of our study is that the population is less ethnically diverse than the general Canadian population. Some measures were derived from a self-reported mailed survey and may be subject to over- or under-reporting. However, commonly biased self-report measures (such as age, weight, and smoking status) all came from the provider, not the patient. Additionally, 8% of the data for medication adherence was missing. Only patients with an available BpTRU™ measurement were included in analysis and because of this, a small number of patients could not be included. Their providers could not obtain a BpTRU™ reading due to the size and shape of the arm. These were very high BMI patients with a "conically" shaped arm.

## Conclusions

Although numerous factors have been shown to be associated with achieving BP targets, our study identified two specific issues that health practitioners should focus on when treating patients who are still not at target. They should emphasize salt-restriction, possibly with referral to a nutritionist, and employ patient-centered strategies to assess and enhance medication adherence.

## Competing interests

The authors declare that they have no competing interests.

## Authors' contributions

WP and BL participated in the conception and design of this study, the analysis and interpretation of results, and drafting and revising the manuscript. FB, MG, RAG, JH, KL, and IM participated in the conception and design of the study, acquisition of data, the interpretation of results, and the revising of the manuscript for intellectual content. FIB, NN, IS, BM, PD took part in the conception and design of the study, the interpretation of the results, and the revising of manuscript for intellectual concept. KVA took part in the analysis and interpretation of the results and the drafting and revising of the manuscript. MSG participated in the interpretation of the results and the revising of the manuscript. All authors have approved the final version.

## Pre-publication history

The pre-publication history for this paper can be accessed here:

http://www.biomedcentral.com/1471-2296/12/86/prepub
